# Hyperdry human amniotic membrane application as a wound dressing for a full-thickness skin excision after a third-degree burn injury

**DOI:** 10.1093/burnst/tkaa014

**Published:** 2020-07-27

**Authors:** Jiro Oba, Motonori Okabe, Toshiko Yoshida, Chika Soko, Moustafa Fathy, Koji Amano, Daisuke Kobashi, Masahiro Wakasugi, Hiroshi Okudera

**Affiliations:** 1 Department of Emergency and Disaster Medicine, University of Toyama, Toyama 930-0194, Japan; 2 Department of Regenerative Medicine, Graduate School of Medicine and Pharmaceutical Sciences, University of Toyama, Toyama 930-0194, Japan; 3 Department of Biochemistry, Faculty of Pharmacy, Minia University, Minia 61519, Egypt; 4 Department of Emergency Surgery, Sakai City Medical Center, Sakai, Osaka 594-8304, Japan

**Keywords:** Hyperdry human amniotic membrane, Third-degree burn, Granulation, Cell migration, Inflammation, Angiogenesis

## Abstract

**Background:**

Severe burn injuries create large skin defects that render the host susceptible to bacterial infections. Burn wound infection often causes systemic sepsis and severe septicemia, resulting in an increase in the mortality of patients with severe burn injuries. Therefore, appropriate wound care is important to prevent infection and improve patient outcomes. However, it is difficult to heal a third-degree burn injury. The aim of this study was to investigate whether hyperdry human amniotic membrane (HD-AM) could promote early granulation tissue formation after full-thickness skin excision in third-degree burn injury sites in mice.

**Methods:**

After the development of HD-AM and creation of a third-degree burn injury model, the HD-AM was either placed or not placed on the wound area in the HD-AM group or HD-AM group, respectively. The groups were prepared for evaluation on postoperative days 1, 4 and 7. Azan staining was used for granulation tissue evaluation, and estimation of CD163, transforming growth factor beta-1 (TGF-β1), vascular endothelial growth factor (VEGF), CD31, alpha-smooth muscle actin (α-SMA) and Iba1 expression was performed by immunohistochemical staining. Quantitative reverse-transcription polymerase chain reaction (PCR) was used to investigate gene expression of growth factors, cell migration chemokines and angiogenic and inflammatory markers.

**Results:**

The HD-AM group showed significant early and qualitatively good growth of granulation tissue on the full-thickness skin excision site. HD-AM promoted early-phase inflammatory cell infiltration, fibroblast migration and angiogenesis in the granulation tissue. Additionally, the early infiltration of cells of the immune system was observed.

**Conclusions:**

HD-AM may be useful as a new wound dressing material for full-thickness skin excision sites after third-degree burn injuries, and may be a new therapeutic technique for improving the survival rate of patients with severe burn injuries.

Highlights

- Hyperdry human amniotic membrane application as a wound dressing for a full thickness skin excision after a third-degree burn injury.
- Hyperdry human amniotic membrane may be useful as a new wound dressing material for full thickness skin excision sites after third-degree burn injuries, and may be a new therapeutic technique for improving the survival rate of patients with severe burn injuries.

## Background

Severe burn injuries render the host susceptible to bacterial infection because of the large skin defects that are created. Burn wound infection often causes systemic sepsis and severe septicemia, resulting in an increase in mortality among patients with severe burn injuries. It is currently estimated that more than 75% of mortality following severe burn injuries is related to infections. Complication of burn wound infection is considered a serious problem in the case of patients with severe burn injuries. Therefore, appropriate burn wound care is critical to prevent infection and improve patient outcome [1, 2].

Artz’s criteria, and the modified version (Moylan’s criteria), are the most widely used clinical criteria for severe burns [3]. The ratio of burn area to total body surface area (TBSA), which is included in Artz’s criteria, is the most basic prognostic factor and a recommended indicator [4]. The burn depth is used as an indicator of the local severity. The most severe burn is a third-degree burn injury, which is defined as burns resulting in damage to the full thickness of the skin. According to Artz’s criteria, a severe burn is defined as 30% TBSA for second-degree burns or 10% TBSA for third-degree burns. Currently, for the local treatment of patients with severe burns, after general management, debridement is performed as early as possible, as well skin grafting if necessary. However, it is difficult to heal a third-degree burn injury [5].

In general, the current standard treatment of a third-degree burn injury is to perform full-thickness skin excision as early as possible, and to perform skin grafting on the same site. Thus, it is important to manage a full-thickness skin excision site until tissue grafting can be performed. In recent years, there have been many reports about the usefulness of artificial dermis and negative-pressure wound therapy (NPWT) as a covering material for full-thickness skin defect sites [6]. However, since severe burns involve a wide range of injury sites, they are not suitable for the management of local burn wounds because of a large amount of exudate from the wounds, less normal residual skin and vulnerability to infection. There is no consensus in the literature regarding appropriate dressing material for full-thickness skin excisions sites of patients with severe burns until skin grafting is performed. By promoting early granulation tissue on the full-thickness skin excision site, it is possible to perform early skin grafting and wound closure. This works effectively for local infection control. Furthermore, it can improve the survival rate of patients with severe burns.

In this study, we focused on the human amniotic membrane (AM) as a biomaterial dressing to enhance the proliferation of granulation tissue on the full-thickness skin excision site after a third-degree burn injury. AM functions as a scaffold to repair tissue [7, 8], and has an anti-inflammatory effect, antibacterial properties, moisture retention characteristics, low antigen level and is inexpensive [9–12]. Previously, we have characterized the cells derived from AM [13–15]. Although fresh AM (fAM) has previously been reported to be a useful covering material for trauma and burn injuries over a long duration [16], it is difficult to immediately obtain fAM when needed; its storage and management are also complicated. To resolve these problems, we previously developed and reported on hyperdry human amniotic membrane (HD-AM) [17]. HD-AM retains various characteristics of fAM and is prepared by obtaining AM by Cesarean section; further drying the tissue under far infrared ray irradiation, reduced pressure, and intermittent microwave irradiation; and then sterilizing the tissue using gamma-ray irradiation. HD-AM can be used when necessary; its storage and handling are simple. It has previously been used as a clinical research tool in various fields and has been shown to be safe for treatment in humans [18–20]. Furthermore, HD-AM still contains biological substances, such as transforming growth factor beta-1 (TGF-β1), interleukin (IL)-8, platelet-derived growth factor (PDGF), vascular endothelial growth factor (VEGF), endothelial growth factor (EGF), IL-6 and IL-10. TGF-β1 exhibits strong migration activity against fibroblasts and macrophages to promote granulation. IL-8 is a cell migratory cytokine for neutrophils.

When used with allogenic transplantation it presents few immunological problems, because the AM, which separates the mother from the fetus, has low antigenicity (Human Leukocyte Antigen (HLA)-DR DR negative, CD59 positive), and Major Histocompatibility Complex (MHC) Class II of human AM is negative and weakly positive for MHC Class I. Moreover, it is unlikely to cause immunological problems because HD-AM has no viable cells due to the hyperdry process.

When using HD-AM as a wound dressing for full-thickness skin excision sites after a third-degree burn injury, granulation tissue growth may be achieved early due to the function of cytokines present in HD-AM and its scaffolding function and induction of various cytokines and chemokines. Moreover, there is also a possibility of enhancing the local immune action. In this study, we created an experimental animal model of a third-degree burn injury in mice. This study histologically, immunohistochemically and genetically examined whether HD-AM could be a promising therapeutic tool for full-thickness skin excision sites after a third-degree burn injury, as we aimed to develop an innovative treatment technology for improving the survival rate of patients with severe burn injuries.

## Methods

### Ethical Statement and informed consent

All experimental procedures, including the use of human AM, were performed according to the study protocol that was reviewed and approved by the Ethical Review Board of the University of Toyama (Reviewed and Evaluated as Case No.44) and Takaoka City Hospital (Reviewed and Evaluated as titled “Supply of amnion for basic studies, the treatment and the surgical repair of refractory ocular surface diseases using the HD-AM”), in accordance with the guidelines of the Declaration of Helsinki.

### HD-AM preparation

HD-AM preparation was prepared as described previously [17, 18, 21], with the following modifications. AMs were placed on cooking sheets (Toyo Aluminum Ekco Products, Tokyo, Japan) and dried sequentially as follows: (1) under the first vacuum (approximately 0.5–0.6 kPa); (2) with far infrared rays using a 0.4 kW heater to keep the chamber at 50°C; (3) with 0.1 kW microwave irradiation (after the air pressure was increased from 0.4 to 4.6 kPa) for 3 minutes; and (4) under the second vacuum (which again decreased the air pressure to less than the first vacuum on the wet sample) using a drying device (Sakura Seiki Co., Tokyo, Japan). After several repetitions of this cycle of air pressure changes, the samples were dried completely but not frozen. Following this drying treatment, the boiling temperature of the AM samples was decreased to approximately 30°C at 4.6 kPa. For sterilization, the packages of HD-AM underwent gamma irradiation (25 kGy). Following this, these packages were stored safely at 4°C for further use.

### Animals

Eight-week-old male Crl: SD-1 Institute of Cancer Research (ICR) mice (body weight 32–37 g) were purchased from Japan SLC (Hamamatsu, Japan). All mice were bred under specific pathogen-free conditions and kept under standard laboratory conditions at the Life Science Research Center, University of Toyama. Standard feed and water were supplied by *ad libitum*. The research protocol was approved by the Ethical Committee of Animal Experimentation, Toyama University (A2017MED-44, A2019MED-33).

### Creation of the experimental mouse model

**Figure 1.**
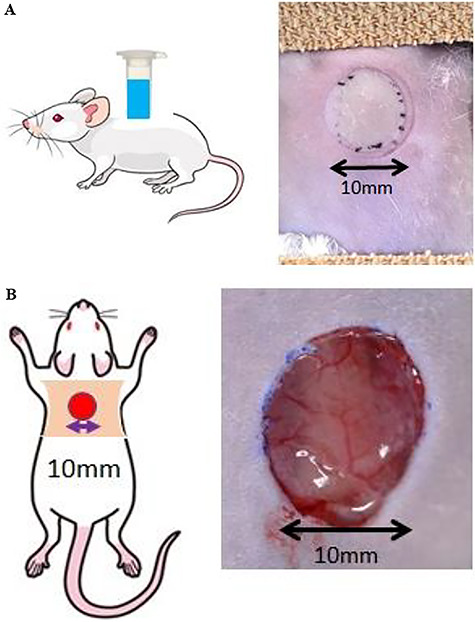
Experimental mouse model. The dorsal skin area (20% total body surface area) was then exposed to hot water (90°C, 10 seconds) in the 1.5 ml reaction tube, which were modified at the bottom to induce a third-degree burn injury. **(a)** Left: scheme of an experimental mouse with a third-degree burn injury. A third-degree burn injury was made at the back of a mouse with a wound 10 mm in diameter. Right: a photograph of the wound site in the mouse model immediately after a third-degree burn injury. Injured skin turned white. **(b)** Left: scheme of an experimental mouse with full-thickness skin excision site. Right: a photograph of the wound site in the mouse model immediately after full thickness skin excision

The mice were anesthetized by intraperitoneal injection of an anesthetic consisting of 3 mg/kg of medetomidine chloride, 4 mg/kg of midazolam, and 5 mg/kg of butorphanol tartrate sodium pentobarbital. Their dorsal hairs were clipped and depilated with a hair removal cream. The dorsal skin area (20% TBSA) was then exposed to hot water (90°C, 10 seconds) in a 1.5 ml reaction tube (Greiner Bio-One, Austria) that was modified at the bottom to induce a third-degree burn injury [22]. Immediately after the burn, the full-thickness skin at the same site was excised and used as an open wound for examination of the wound healing process (Figure 1).

### Confirmation of a third-degree burn injury

In order to judge whether the created burn model was appropriate as a third-degree burn injury, the correlation between burn depth and the post-burn injury progress was confirmed. Histological evaluation of the epidermis, dermis and subcutaneous tissue at the injury site was performed. Furthermore, a histological evaluation of the presence or absence of epithelialization and the dermis layer at the site where the epidermal layer sloughed off 7 days after the third-degree burn injury was performed.

A 10 mm diameter of the wound area was limited as the treatment area at the back of the mouse. A circular burn area 10 mm in diameter is equivalent to 3% of the TBSA in mice. It is not suitable to evaluate systemic changes, such as severe burns, in experimental models, but is sufficient for evaluating third-degree burn sites. In this experiment, our aim was to determine whether HD-AM is suitable for making a wound bed (granulation) before skin grafting.

### Application of HD-AM for the experiment

**Figure 2.**
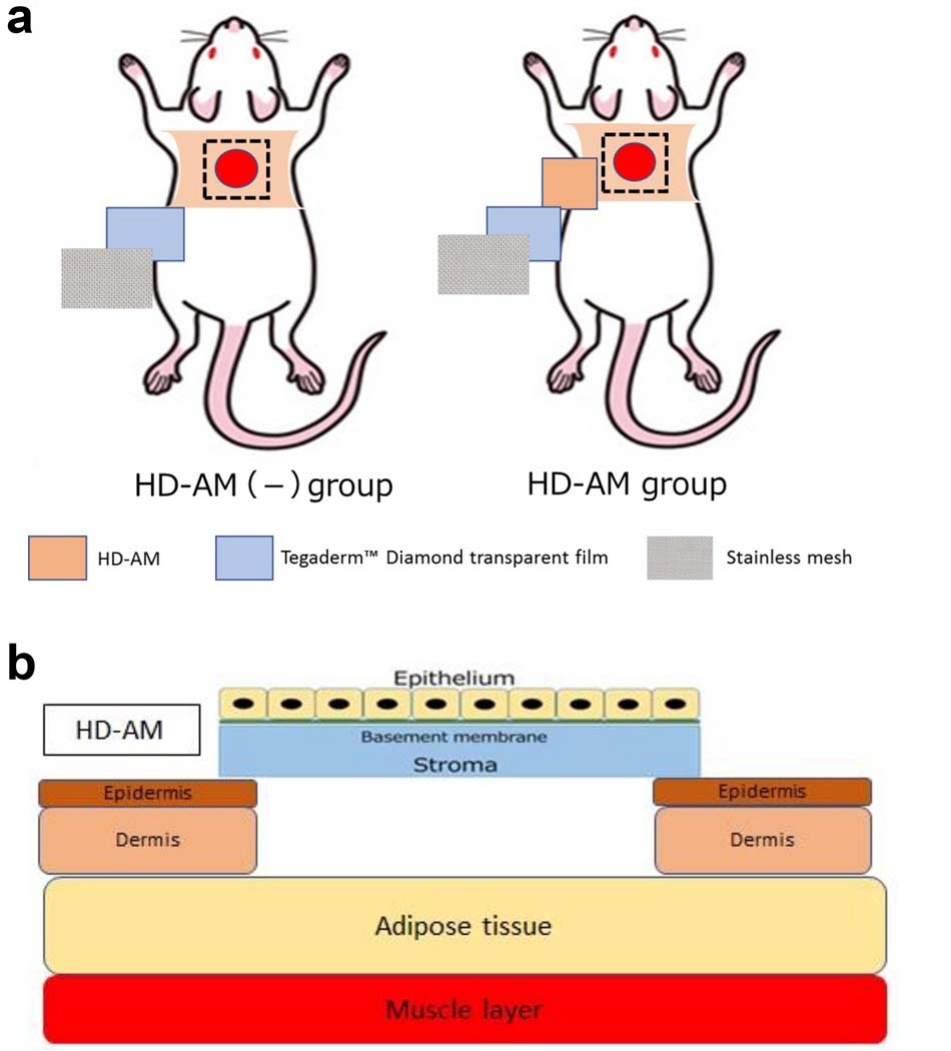
Application of HD-AM. **(a)** Experimental groups. Left: HD-AM was not used in the control group (HD-AM (-) group), in which only the polyurethane foam dressing and stainless mesh were used to cover the injured site. Right: HD-AM was placed over the full-thickness skin excision site with the epithelial side facing upwards (HD-AM group). **(b)** Scheme of full-thickness skin excision site covered with HD-AM. *HD-AM* hyperdry human amniotic membrane

Human AM is composed of three major layers: a single epithelial layer, a thick basement membrane and a vascular stromal layer. HD-AM was cut into a 15 × 15 mm piece and placed on the wound area with the epithelial side facing upwards (the HD-AM group). Meanwhile, HD-AM was not used in one group (the HD-AM (-) group), which formed the control group (Figure 2). The wound was covered with a polyurethane foam dressing, Tegaderm™ Diamond transparent film® (3M Deutschland GmbH Health Care Business). Each group comprised 4–6 mouse models that were prepared separately on postoperative days (PODs) 1, 4 and 7 for evaluation. The wound site was protected with a stainless mesh (0.06 mm Φ, 150 mesh (m/s)) cover to prevent the wound site from losing the HD-AM dressing or the wound dressing materials being affected by mice activity.

### Evaluation of each tissue

**Figure 3.**
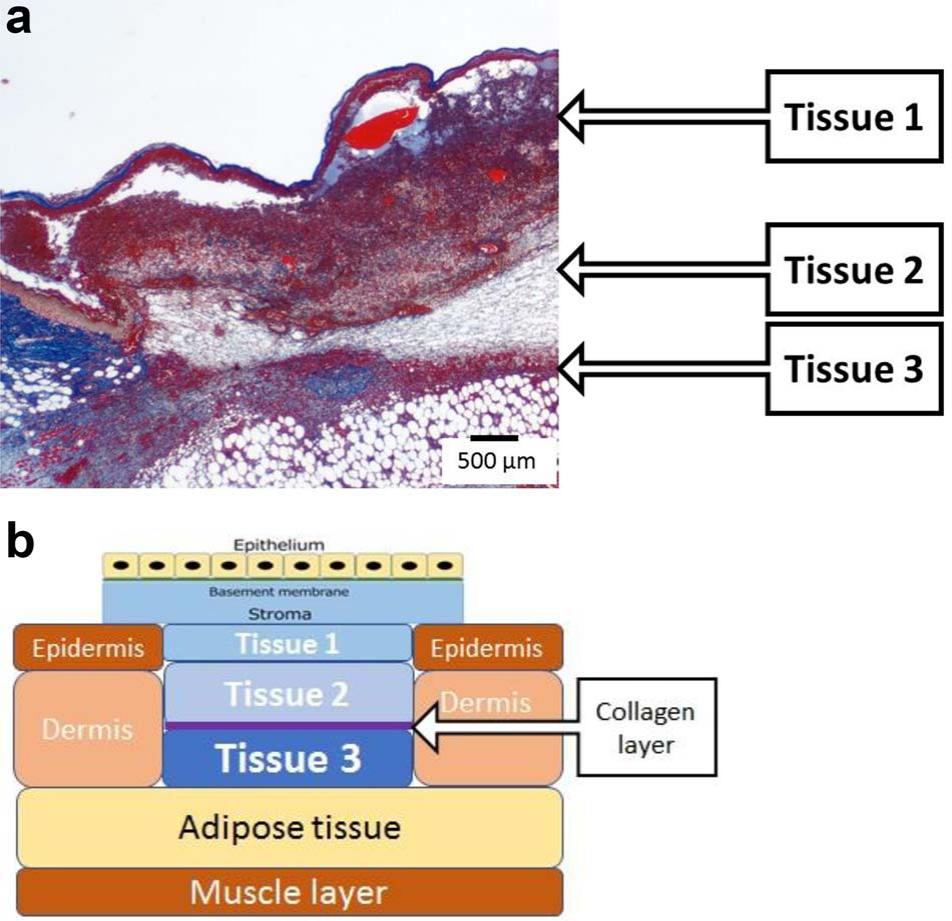
Tissue of the HD-AM group (POD 4). The full-thickness skin excision site after a third-degree burn was divided into regions with Tissue 1 directly below HD-AM, Tissue 2 above the collagen fiber layer and Tissue 3 below the collagen fiber layer. **(a)** A section of the full-thickness skin defect site was observed histologically using azan staining (×25). **(b)** Schematic diagram of the full-thickness skin defect site: a collagen layer has separated Tissue 2 from Tissue 3. *HD-AM* hyperdry human amniotic membrane, *POD* postoperative day

By using Azan staining, a thin collagen fiber layer extending from the normal skin around the full-thickness skin excision site was observed on POD 1. When we observed the HD-AM group over time, morphological changes were observed in cells that invaded the layers above and below the thin collagen fiber layer and the layer directly below the HD-AM. Therefore, as shown in Figure 3, the full-thickness skin excision site after a third-degree burn was divided into regions with Tissue 1 directly below HD-AM, Tissue 2 above the collagen fiber layer, and Tissue 3 below the collagen fiber layer (Figure 3).

### Measurement of the granulation tissue at the full-thickness skin excision site

**Figure 4.**
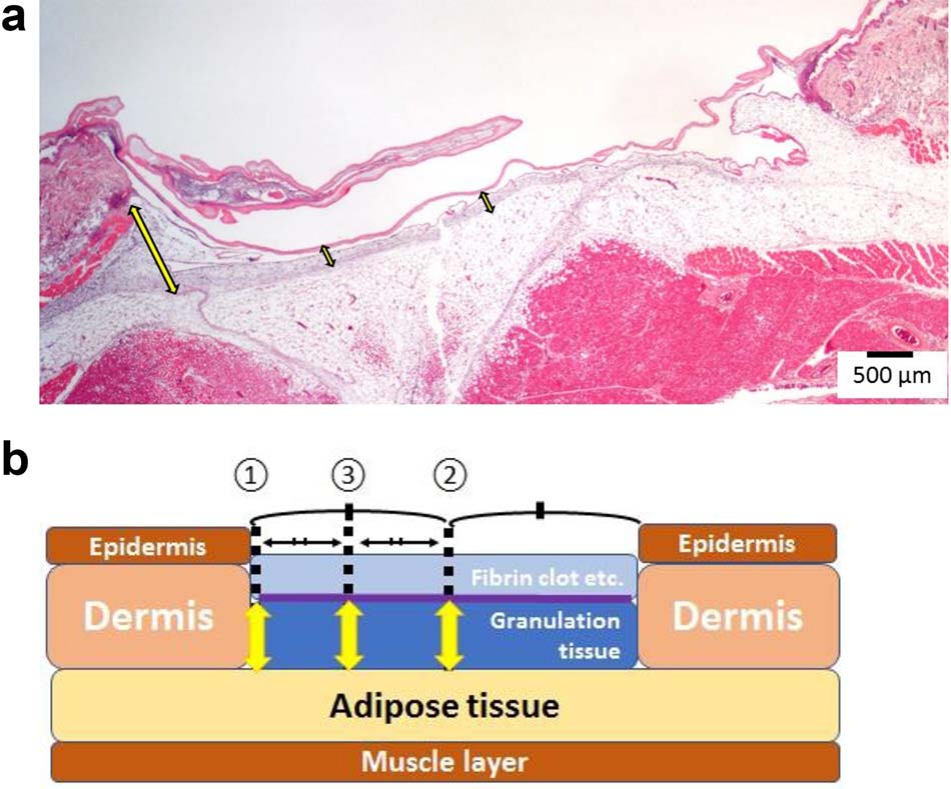
Measurement the thickness of the granulation tissue layer. The thickness of the granulation tissue formed in Tissue 3 was measured using the Olympus CellSens imaging program. **(a)** A continuous section of the HD-AM group was observed histologically using H&E staining (x25) at each time point. **(b)** Schematic diagram of measuring points about the thickness of granulation tissue (Tissue 3). The measurement sites were one end ① the center from both ends ② and the middle point from the center to one end ③ of the injured area. The average values of the three points were compared. *HD-AM* hyperdry human amniotic membrane, *H&E* hematoxylin and eosin

Azan staining was used to clarify the distinction between collagen fibers and fibrin. The thickness of the granulation tissue formed in Tissue 3 was measured using the Olympus CellSens imaging program (version 1.7; Olympus Co., Tokyo, Japan). The measurement sites were three points, as shown in Figure 4, and the average values were calculated and compared. The measurement sites were one end, the center from both ends, and the middle point from the center to one end (Figure 4). The average values of the three points were compared.

### Histological and immunohistochemical staining

All tissues newly formed at the full-thickness skin excision site on PODs 1, 4 and 7 were collected from each mouse. These resected samples were dehydrated in a series of graded ethanol baths and a xylene bath, prior to being immersed in paraffin wax. The samples were then embedded in paraffin molds and sliced into 4 μm-thick sections that were mounted on glass slides and stained with hematoxylin and eosin (H&E) and azan. Immunohistochemical staining was performed for the specimens on PODs 1, 4 and 7. The specimens were incubated overnight with a primary antibody against either CD163 (1:500; Abcam, Cambridge, UK), TGF-β1 (20 μg/ml, Abcam), VEGF (10 μg/ml, Abcam), CD31 (1:500, Abcam), alpha-smooth muscle actin (α-SMA) (1:800, 4 μg/ml, Abcam) or Iba1 (1:200, 1.5 μg/ml, Abcam). The sections were treated with biotinylated anti-rabbit, anti-mouse or anti-goat IgG secondary antibody (Nichirei Biosciences Inc., Tokyo, Japan). A color-developing agent was obtained by treatment with a DAB kit (Nichirei Biosciences Inc.). The stained tissues were examined using a Leica DMRBE microscope (Leica, Wetzlar, Germany) and digital camera DP73 (Olympus Co.).

### Measurement of messenger RNA by quantitative reverse-transcription polymerase chain reaction

To extract messenger RNA (mRNA) from the granulation tissue on the full-thickness skin excision site, target sites were selectively collected from each sample. Total mRNA was extracted from the specimens using Isogen II (Nippon Gene Co. Ltd., Tokyo, Japan), according to the manufacturer’s instructions. Aliquots of 3 μg of total mRNA were treated with deoxyribonuclease I (DNase I, Sigma-Aldrich, Inc., Tokyo, Japan) at room temperature for 15 minutes. cDNAs were synthesized using 500 ng of DNase I-treated mRNA, using a ReverTra Ace qPCR RT Kit (Toyobo Co., Ltd, Osaka, Japan). Gene expression was quantitatively assessed through real-time RT-PCR analyses using Brilliant SYBR Green qRT-PCR Mix (Stratagene; Agilent Technologies Japan, Ltd., Japan) with the Mx3000P quantitative polymerase chain reaction (qPCR) system (Stratagene; Agilent Technologies Japan, Ltd, Japan). The following primers for the following proteins were used for qRT-PCR: growth factors (TGF-β1 [23], VEGFA [24], α-SMA [25], and PDGF [26]), cell migration chemokine (CXCL-5 [27]), representative marker for anti-inflammatory M2 macrophages (CD163 [28]), anti-inflammatory cytokines (IL-10 and IL-6 [29]), inducible nitric oxide synthase (iNOS) [30], vascular endothelial marker (CD31 [31]), and cyclooxygenase-2 (COX-2) [32]. qRT-PCR was performed on PODs 1, 4, and 7 in triplicate. The expression level of each mRNA was normalized to glyceraldehyde-3-phosphate dehydrogenase as an internal control. The tissue collected immediately from the full-thickness skin excision site after third-degree burn injury was designated POD 0, and the expression of each group was expressed relative to the expression at POD 0. The primer sequences and each annealing temperature are listed in Table 1.

**Table 1:** List of polymerase chain reaction primer sequences

	Forward primer	Reverse primer	Annealing temp (°C)
$\mathrm{TGF}\hbox{-} \beta 1$	CAACAATTCCTGGCGTTACI CIGG	GAAAGCCTCGTATTCCGTCTCCTT	60
VEGFA	CACAGCAGATGTGAATGCAG	TGGCGGGGACTATGAAGGT	60
a-SMA	CCCCTGAAGAGCATCGGACA	TTTACACGTCTGCGGATCTT	60
PDGF	TGTGCCCATTCGCAGGAAG	GAGGTATCTCGTAAATGACCGTC	56
CXCL-5	TTCATGAGAAGGCAATGCTG	CCCAGGCTCAGACGTAAGAA	56
iNOS	GTTCTCAGCCCAACAATACAAGA	GTGGACGGGTCGATGTCAC	60
IL-6	CCTCTGGTCTTCTGGAGTACC	ACTCCTTCTGTGACTCCAGC	55
CD163	GGACAGATCTGGGTGAAGA	ATCCCTGCTGTGGGTACAAG	56
IL-10	ATAACTGCACCACCTTCCCA	GGGCATCAI CICTACCAGGT	55
CD31	ATGCTCCTGGCTCTGGGACTCACG	GTGCTGAGACCTGCTTTTCGAGGT	60
COX-2	CAGACAACATAAACTGCGCCTT	GATACACCTCTCCACCAATGACC	53
GAPDH	TGTGTCCGTCGTGGATCTGA	TTGCTGTTGAAGTCGCAGGAG	57

### Statistical analysis

All values were expressed as mean ± standard error. Using statistical software (SPSS Statistics version 25 for Windows; IBM, Tokyo, Japan), comparisons between different groups were made using either one-way or two-way analysis of variance. When *p* < 0.05, differences were considered significant.

## Results

### Validation of the third-degree burn injury model

In the preliminary experiment, the test was performed by exposing the backs of the mice to hot water (90°C) for 10 seconds, within 10 mm diameter. The burn depths did not change, even after third-degree burns (immediately, 30 minutes, 1 hour, 2 hours and 6 hours after burn injury).

Immediately after exposing mice to 90°C water for 10 seconds, the skin color of the exposed area became fair but that of the neighboring area did not change. After 7 days, the affected area changed color from fair to brown. However, histologically, the resected specimen showed no changes depicting thermal damage to the surrounding area.

**Figure 5.**
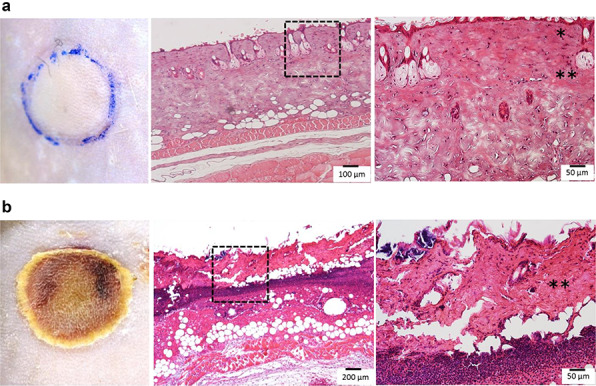
Macro- and microscopic photographs of the third-degree burn injury. **(a)** Photographs were taken immediately after a third-degree burn injury. Left: a photograph of the wound site where the injured skin turned white. Middle and right: the histology of the injured area. The epithelial layer was fluffy and the dermis was like hyaline. The right image (×200) shows an enlargement of the elongated dotted frame in the middle photograph (×100). The right photograph shows tissue edema, necrosis and some loss in the epidermis; collagen fibers are difficult to distinguish from each fiber due to hyaline-like degeneration. (^*^epidermis, ^**^dermis) **(b)** Images taken 7 days after the third-degree burn injury. Left: the injured site was covered with scabs. Middle and right: the histology of the injured area. The necrosis area in the dermis was enlarged. The right image (×200) shows an enlargement of the elongated dotted frame in the middle photograph (×50). No epithelial layer was observed; a part of the dermis was destroyed and cell debris had accumulated (^*^^*^dermis)

A section of the injured site was observed histologically using H&E stain. Tissue edema, necrosis and some losses in the epidermis were observed. Collagen fibers, such as hyaline fibers, in the dermis were damaged (Figure 5a), and the epidermis did not regenerate. Histologically, the dermis layer was damaged and hollowed out and cells were infiltrated, but granulation was not observed (Figure 5b). Based on the above findings, this experimental model was designated as a third-degree burn injury model.

### Granulation tissues evaluation

**Figure 6.**
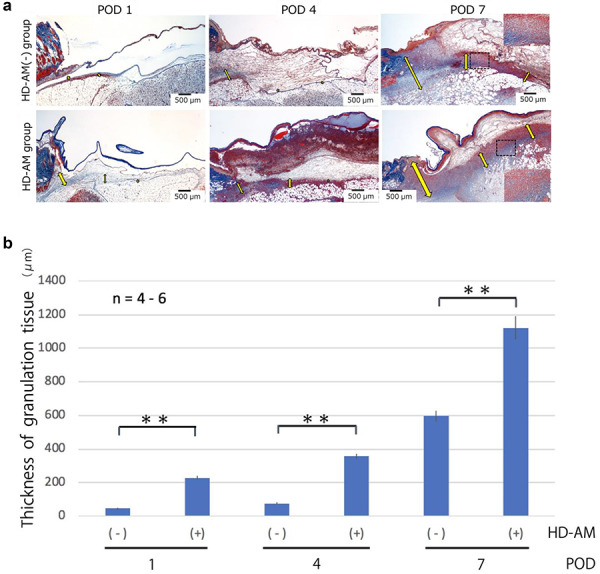
Evaluation of the thickness of granulation tissues. **(a)** Representative microscopic photos of each group at different time points using Azan staining (×25). Scale bars, 500 μm. Tissue 2 became a fibrin clot on POD 7. Fibroblast accumulation and angiogenesis were observed in Tissue 3. However, in Tissue 1, only the accumulation of migratory cells was observed. Therefore, Tissue 3 was judged as the formation area of granulation tissue. The thickness of the granulation tissue is indicated with a yellow arrow. The HD-AM (-) group (on POD 1) does not have any arrows because the granulation tissue was too thin. The rectangular (×200) in the dotted frame area was enlarged. **(b)** A graph was used to compare the thickness measured at three time points in the formed granulation tissue. Bars represent mean ± standard error. The HD-AM group showed a significantly thicker granulation tissue on PODs 1, 4 and 7 than the control (*n* = 4–6,^*^^*^p < 0.01). The HD-AM group had a significantly thicker granulation tissue layer on PODs 1, 4 and 7 than the HD-AM (-) group. *HD-AM* hyperdry human amniotic membrane, *POD* postoperative day

Over time, Tissue 1 gradually became thinner and it was the thinnest on POD 4. Tissue 2 became thinner over time, and Tissue 3 became thicker. As a result, Tissue 2 became a fibrin clot. Since Tissue 3 showed fibroblasts and angiogenesis, it was judged that granulation was promoted in Tissue 3 (Figure 6a). Tissue 3 was judged to be a granulation tissue, and the thickness of the same site was measured in each group and examined over time (on PODs 1, 4 and 7). The HD-AM group and the HD-AM (-) group were compared; the HD-AM group had a significantly thicker granulation tissue layer at any time point of PODs 1, 4 and 7 (*n* = 4–6, *p* < 0.01) (Figure 6b).

### Cell infiltration evaluation

**Figure 7.**
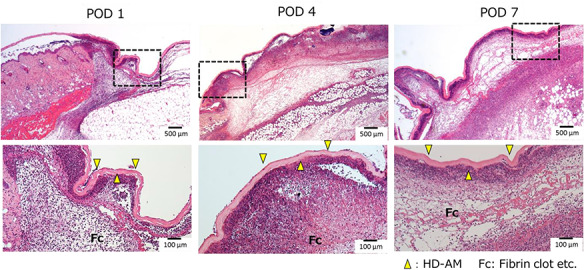
Cell infiltration under HD-AM (Tissue 1). Cell infiltration was observed directly below the HD-AM using H&E staining (×25), (Top). The Bottom images (×100) show an enlargement of the elongated dotted frame in the top images (×25). The peak of cell infiltration was observed on POD 4. On POD 7, in Tissue 1, cell infiltration was decreased but there was an active formation of granulated tissue in Tissue 3. Yellow arrows: HD-AM. *HD-AM* hyperdry human amniotic membrane, H&E hematoxylin and eosin, *POD* postoperative day

As shown in Figure 7, in the HD-AM group, cell infiltration was observed in Tissue 1 (directly below HD-AM) and Tissue 3 at all time points. Infiltrating cells were neutrophils, macrophages and lymphocyte lineage. Cell infiltration of Tissue 1 (directly below HD-AM) was continuously observed up to POD 7. However, it was the most common on POD 4, but decreased thereafter.

### Evaluation of mRNA expression by qRT-PCR

#### Expression of growth factors (TGF-β1, VEGF, α-SMA, PDGF), cell migration chemokine (CXCL-5), vascular endothelial marker (CD31) and COX-2

**Figure 8.**
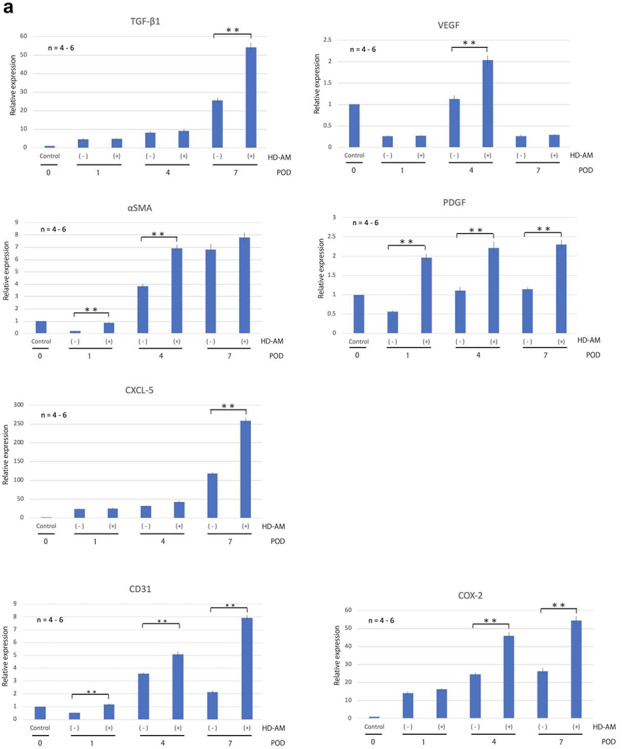

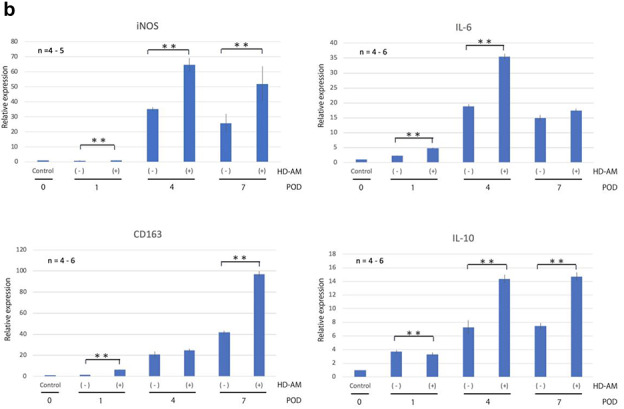
qRT-PCR was performed on PODs 1, 4, and 7 in triplicate. The expression level of each mRNA was normalized to glyceraldehyde-3-phosphate dehydrogenase as an internal control. The tissue collected immediately from the full-thickness skin excision site after third-degree burn injury was designated POD 0, and the expression of each group was expressed relative to the expression at POD 0. **(a)** The expression of mRNA of growth factors (TGF-β1, VEGF, α-SMA, PDGF), cell migration chemokine (CXCL-5), vascular endothelial marker (CD31) and COX-2. **(b)** The expression of mRNA in relation to proinflammatory and anti-inflammatory markers. Bars represent mean ± standard error. The expression of each gene in different groups was expressed relative to that of the control group on POD 0 (*n* = 4–6, ^*^^*^p < 0.01). *TGF*-β*1*: transforming growth factor beta-1, *VEGF* vascular endothelial growth factor, α-*SMA* alpha-smooth muscle actin, *PDGF* platelet-derived growth factor, *POD* postoperative day. *HD-AM* hyperdry human amniotic membrane, *qRT-PCR* quantitative reverse-transcription polymerase chain reaction, *iNOS* inducible nitric oxide synthase, *IL* interleukin, *mRNA* messenger RNA

As shown in Figure 8a, the expression of TGF-β1 increased over time, regardless of the presence or absence of HD-AM. On POD 7, the expression of TGF-β1 in the HD-AM group was significantly (*p* < 0.01) higher than in the HD-AM (-) group. VEGF expression was highest on POD 4, and significantly (*p* < 0.01) higher in the HD-AM group than in the HD-AM (-) group. The expression of α-SMA tended to increase over time, regardless of the presence or absence of HD-AM. On PODs 1 and 4, the expression of α-SMA in the HD-AM group was significantly higher than that in the HD-AM (-) group. The expression of α-SMA was not significantly different between the two groups on POD 7. PDGF expression did not change significantly over time in the HD-AM (-) group, but its expression level decreased on POD 1. Conversely, in the HD-AM group, the expression of PDGF continued to be approximately twice that of POD 0 from POD 1 to 7. Furthermore, the expression was significantly (*p* < 0.01) higher than that of the HD-AM (-) group on PODs 1, 4 and 7. The expression of the cell migration chemokine (CXCL-5) increased over time, regardless of the presence or absence of HD-AM. On POD 7, the expression level of the HD-AM group was about twice that of the HD-AM (-) group. CD31 expression was highest on POD 4 in the HD-AM (-) group. Conversely, the expression of CD31 in the HD-AM group was significantly (*p* < 0.01) higher than that in the HD-AM (-) group on POD s1, 4 and 7, and the expression level tended to increase over time. COX-2 expression tended to increase over time in each group. The expression of COX-2 in the HD-AM group was significantly (*p* < 0.01) higher than that in the HD-AM (-) group on PODs 4 and 7.

#### Expression of mRNA for proinflammatory and anti-inflammatory markers

Figure 8b shows that the expression of iNOS was significantly (*p* < 0.01) higher in the HD-AM group than in the HD-AM (-) group on PODs 4 and 7. IL-6 expression was significantly (*p* < 0.01) higher in the HD-AM group than in the HD-AM (-) group on PODs 1 and 4. The expressions of iNOS and IL-6 tended to decrease on POD 7 after peaking on POD 4. CD163 and IL-10 expressions tended to increase over time in each group, and their expressions were higher in the HD-AM group than in the HD-AM (-) group on PODs 1, 4 and 7. CD163 expression was significantly (*p* < 0.01) higher in the HD-AM group on POD 1 and 7, whereas IL-10 was significantly (*p* < 0.01) higher in the HD-AM group on PODs 4 and 7.

### Immunohistochemical staining

**Figure 9.**
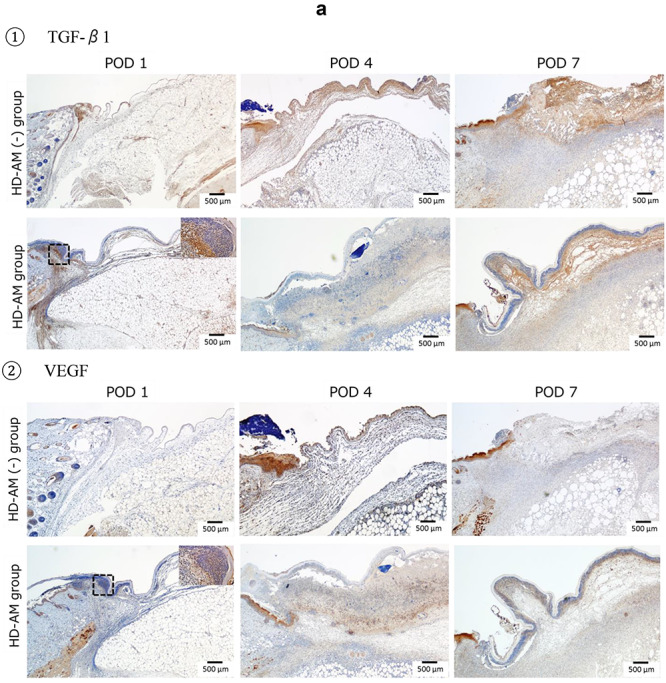

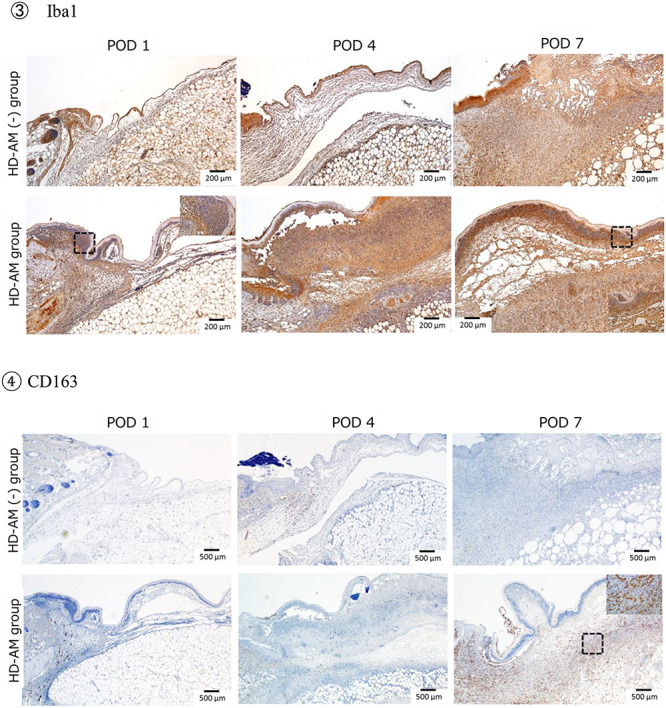

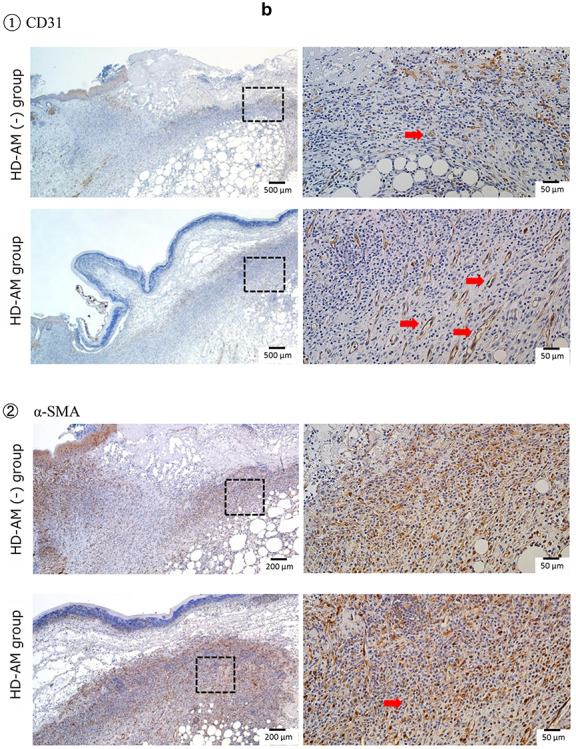
Immunohistochemical staining. **(a)** Immunohistochemical staining of infiltrated cells under HD-AM (each group on PODs 1, 4 and 7). ①: TGF-β1 (×25), ②: VEGF (×25), ③: Iba1 (×50), and ④: CD163 staining (×25). The square (×400) in the dotted frame area was enlarged. Notice the TGF-β1-positive cells aggregated in Tissues 1 and 2. VEGF-positive cells were observed in Tissue 2 on POD 4 in the HD-AM group only. More Iba1-positive cells were located in Tissues 1 and 2 at every time point in the HD-AM group than the HD-AM (-) group. CD163-positive cells were observed in Tissue 3 only on POD 7. **(b)** Analysis of constitution of granulation tissue with immunohistochemistry. ①: CD31 staining: comparison of HD-AM group and HD-AM (-) group on POD 7 for localization of CD31-positive cells; angiogenesis (red arrow) was more frequently observed in the HD-AM group than in the HD-AM (-) group. The focused part of the dotted frame on the left photograph (×25) is enlarged and is shown in the right photograph (×200). ②: α-SMA staining: comparison of the HD-AM and HD-AM (-) groups on POD 7 for localization of α-SMA-positive cells; fibroblasts (red arrow) were more frequently observed in the HD-AM group than in the HD-AM (-) group. The focused part of the dotted frame on the left photograph (×25) is enlarged and is shown in the right photograph (×200). *HD-AM* hyperdry human amniotic membrane, *TGF*-β*1* transforming growth factor beta-1, *VEGF* vascular endothelial growth factor, *POD* postoperative day, α-*SMA* alpha-smooth muscle actin

In the HD-AM group, TGF-β1, VEGF and Iba1 were observed to be expressed in Tissue 1 (directly below HD-AM) from POD 1. Over time, they expressed diffusely and highly in Tissue 2 (in fibrin clot) and Tissue 3 (granulation tissue) (Figure 9a: ①–③). VEGF was abundantly expressed at any part in the HD-AM group on POD 4. CD163 was hardly observed in the HD-AM group on POD 4 but was highly expressed in Tissue 3 (granulation tissue) on POD 7. However, it was not observed at all in Tissue 1 (directly below HD-AM) (Figure 9a: ④. Comparison of the expression of Iba1 (a marker for M1 and M2 macrophages) and CD163 (a marker for M2 macrophages) in the HD-AM group on POD 7 revealed the subclass localization of macrophages (Figure 9a: ③,④).

CD31 was highly expressed, indicating angiogenesis in the granulation tissue of the HD-AM group on POD 7 (Figure 9b: ①, red arrow). However, no expressions of CD31 were observed in the tissue directly below HD-AM on POD 7. α-SMA was expressed in many fibroblasts of the granulation tissue in the HD-AM group on POD 7 (Figure 9b: ②, red arrow). In addition, similar to CD31, no expressions of α-SMA were observed in the tissue directly below HD-AM on POD 7.

## Discussion

A method for creating severe burn injury mouse models by exposing mice to hot water had been reported previously [22], but special equipment for fixing the mouse was needed. In this study, a modified reaction tube was used on the back. In the preliminary experiment, the depth of the burn was evaluated over time by changing the temperature of the hot water and the exposure time. A repetition of this process helped to sufficiently evaluate whether the burn was appropriate. The test was performed by exposing the backs of the mice to hot water at 90°C for 10 seconds, on a circular area of skin 10 mm in diameter. It was found that the burn depth did not change even after third-degree burns (immediately, 30 minutes, 1 hour, 2 hours and 6 hours after burn injury). Preliminary experiments confirmed that this method was inexpensive and stable, and that burns of various depths could be made.

In our experimental burn mouse model, the burn depth reached the entire layer of subcutaneous tissues immediately after the injury, and no epithelialization was observed until 7 days after the injury. Therefore, we determined that this mouse model was an appropriate model for third-degree burn injuries frequently observed in clinical practice. Therefore, we used this model for evaluating wound healing at the full-thickness skin excision site after third-degree burn injury. A number of histological, immunohistochemical and molecular procedures were used in the evaluation in this study.

Human AM graft is one of the most medically accepted and widely used biomaterials in burn wound healing treatment since 1910 [33, 34]. However, the difficulties in processing, transporting and storing thin sheets of AM have limited its clinical applications. Rahman *et al*. [35] reported that dissolved amniotic membrane with *Aloe vera*, which has been used in treating any injury, possesses potent wound healing properties for second-degree burns in animal models. AM and its extraction were useful for wound healing, which involves cell proliferation, increased angiogenesis, collagen production and epithelialization. However, in severe ocular burns [36] and third-degree burns, no epithelialization occurs because no epithelial stem cells are available to undergo epithelialization.

In this study, we focused on promoting granulation formation (transplant bed formation) to increase the success rate of skin transplantation.

There are two assessments of the wound healing process. The first method is to calculate the wound closure rate from the wound area and evaluate epithelialization. The second method is to measure granulation thickness histologically and evaluate granulation hyperplasia. This study does not seek the efficiency of wound closure by the wound healing process. Severe burn as a third-degree burn is not able to undergo epithelialization, just as in ocular burns, using amnion [36]. No stem cells were available for development of epithelium and/or cornea for both injuries. In general, the current standard treatment of a third-degree burn injury is to perform full-thickness skin excision as early as possible, and to perform skin grafting on the same site.

In burn wound healing, it is most important to form a qualitatively good granulation tissue (transplant bed). In our study, we focused on whether the formed granulation tissue is qualitatively good. Qualitative evaluation of good granulation tissue requires accumulation of inflammatory cells (macrophages, neutrophils, lymphocytes etc.), fibroblast migration and proliferation, accumulation of extracellular matrix and angiogenesis [37, 38]. In addition, the acute wound healing process consists of three phases: the inflammatory phase, the proliferative phase and the remodeling phase. When a wound is created, platelets aggregate and cover the wound and various cytokines and cell growth factors are secreted; neutrophils, macrophages and lymphocytes infiltrate the wound as well. In the proliferative phase, epidermal cells, fibroblasts and vascular endothelial cells proliferate, resulting in re-epithelialization and formation of granulation tissue. After that, the remodeling phase starts, in which scar tissue, once generated, is replaced with normal tissue structure. When the inflammatory phase occurs and completes early, the next proliferative and remodeling phases successfully take over and good wound healing is achieved. However, if the inflammatory phase is interrupted for any reason it persists, making it is impossible for the proliferative phase to begin smoothly. Such wounds are called chronic or intractable wounds [39]. In order for good granulation tissue to format an early phase, it is important that qualitative construction [of what] occurs and that the healing process moves forward appropriately. In the HD-AM group, Tissue 3 (in the granulation tissue) and Tissue 1 (directly below the HD-AM) had inflammatory cell infiltration from POD 1, and more fibroblast proliferation and angiogenesis were observed in Tissue 3 than in the HD-AM (-) group on POD 7. Therefore, in the HD-AM group, qualitatively good granulation was achieved from an early stage.

We will discuss why the factors that define qualitatively good granulation tissue were achieved early in the HD-AM group.

### Effect of HD-AM on the promotion of inflammatory cell infiltration

The stromal side of the AM has higher cell migration and adhesion functions than the epithelial side, and these have been reported to play an important role in granulation tissue regeneration [40]. The HD-AM has been reported to be effective as a scaffold during tissue regeneration, similar to raw or cryopreserved AM. Furthermore, HD-AM still contains biological substances, such as TGF-β, IL-8, PDGF, VEGF, EGF, IL-6 and IL-10. TGF-β1 exhibits strong migration activity against fibroblasts and macrophages to promote granulation [41]. IL-8 is a cell migratory cytokine for neutrophils [42]. We consider that these factors and the function of HD-AM as a scaffold led to early infiltration of more inflammatory cells into Tissue 1 (directly below the HD-AM). Growth factors, such as TGF-β1, VEGF and Iba1, were diffusely expressed on POD 1 in Tissues 1, 2 and 3, as shown by immunohistochemical staining. Therefore, it is considered that secretion from inflammatory cells infiltrating Tissue 1 caused further infiltration and activation of inflammatory cells into Tissue 2 and Tissue 3. It is suggested that the inflammatory cells that infiltrated Tissue 1 were trapped in the HD-AM, and the growth factor secreted from these cells was fixed to the fibrinoid of Tissue 2.

CXCL-5 expression was higher in the HD-AM group than in the HD-AM (-) group on POD 4 and it further increased on POD 7. CXCL-5 is a chemokine with potent neutrophil/monocyte migration and activation ability. It has been reported that neutrophils/monocytes collect at the wound site and play an important role in wound healing and infection prevention [43]. A total of 75% of deaths among patients with severe burn injuries are due to sepsis caused by a wound infection. This may be because cell-mediated immunity declines immediately after the burn, reaching a minimum in 4–7 days. At that time, the systemic infection easily progresses, due to invasion from the local wound [2]. In tissue repair, inflammatory cells, such as neutrophils, macrophages and lymphocytes, play an important role by releasing cytokines and cell growth factors in addition to local immunity and inflammation, such as removal of bacteria and foreign substances [44]. These results suggest that HD-AM may promote the migration of neutrophils/monocytes from the inflammatory phase to the proliferative phase and improve the defense mechanism against the entry of foreign substances.

### Effect of HD-AM on fibroblast migration and proliferation

From the results of the immunohistochemical staining, Iba1 positive cells were diffusely present on POD 1, regardless of the area. In Tissues 1 and 2, they tended to increase most on POD 4 and decrease on POD 7. Iba1 is constitutively expressed in monocytes and macrophages, known to be involved in macrophage activation, and used as a marker for activated macrophages (markers for M1 and M2 macrophages). The decrease in Iba1 positive macrophages after peaking on POD 4 suggests a transition to the next step in the wound healing stage.

Regarding the expression of PDGF, the expression level on POD 1 was lower in the HD-AM (-) group than on POD 0. Conversely, in the HD-AM group, the expression continued to be more than double the expression on POD 0. This indicates that, in the HD-AM group, the expression of PDGF from cells, such as macrophages infiltrating Tissue 1 and PDGF remaining in HD-AM, is more than that derived from platelets by excision of the damaged site immediately after the burn. PDGF has been reported to migrate fibroblasts and macrophages and promote cell proliferation, along with Epidermal growth factor (EGF) and Insulin-like growth factor (IGF), in plasma [45]. We consider that PDGF is one of the substances that promoted the migration of fibroblasts into the granulation tissue in the HD-AM group.

TGF-β1 expression increased over time and was significantly higher in the HD-AM group than in the HD-AM (-) group on POD 7. TGF-β1 proliferates fibroblasts, promotes the synthesis of collagen, elastin, fibronectin etc. and is reported as a wound healing promoter for the dermis [46]. It is thought that the gradual increase in TGF-β1 promoted the migration and proliferation of fibroblasts into the granulation tissue.

From the immunohistochemical staining, in the HD-AM group, α-SMA, which is expressed in the actin fibers in smooth muscle cells in vessel walls, gut wall, myometrium, myoepithelial cells and myofibroblasts, was expressed in the granulation tissue of Tissue 3 on POD 7, and no expression was observed immediately below the HD-AM in Tissue 1. In addition, the expression of α-SMA mRNA in the HD-AM group was significantly higher than in the HD-AM (-) group on PODs 1 and 4, but no significant difference between the two groups was observed on POD 7. These results suggest that the fibroblasts that migrated and proliferated to Tissue 3 early due to PDGF or TGF-β1 do not proliferate randomly. As a result, granulation was completed earlier in the HD-AM group than in the HD-AM (-) group.

### Effect of HD-AM on angiogenesis

In the process of granulation, the interaction between fibroblasts and vascular endothelial cells via VEGF plays an important role. Moreover, VEGF is important for vasculogenesis and angiogenesis. Various cytokines, such as VEGF, and growth factors act to proliferate vascular endothelial cells, stabilize blood vessels and form a functional vascular network for wound healing. Fibroblasts whose proliferation is promoted by TGF-β1 and PDGF also enhance the secretion of VEGF by these cytokines [47]. VEGF expression was transiently significantly higher in the HD-AM group on POD 4. In the HD-AM group, Iba1 positive cells were diffusely present on POD 1, regardless of the area. Iba1 promotes the proliferation of vascular smooth muscle cells and lymphocytes [48], suggesting that HD-AM is involved in angiogenesis immediately after the burns. We consider that, under the influence of HD-AM, VEGF secretion was increased due to the effects of various related promoters that enhanced macrophage and fibroblast migration and proliferation from an early phase. In addition, transient enhancement of VEGF on POD 4 induced angiogenesis. As a result, on POD 7, a new blood vessel of CD31 positive, which is a vascular endothelial marker, was observed as a blood vessel image that markedly ran from the basal side toward the epithelium. 

It is reported that overexpression and prolonged proliferation of VEGF causes scar formation and fibrosis [49–51]. In addition, there are reports that VEGF expression decreases in mice with delayed wound healing, whereas VEGF expression is observed in wounds in normal mice. The enhanced transient expression of VEGF may be related to the wound healing and scar suppression effects of HD-AM [52, 53]. Further research in this area is required to elucidate the precise nature of the mechanism.

It was reported that increased production of prostaglandin E2 (PGE2) is involved in angiogenesis at the site of the wound inflammation [54]. However, the direct measurement of PGE2 is difficult because its half-life is very short; we therefore measured the levels of COX-2, which is required for the production of PGE2, and used these measurements as a surrogate marker. This supports our results that COX-2 expression was significantly higher in the HD-AM group on PODs 4 and 7.

### Effect of HD-AM on the acute wound healing process

Macrophages can be classified into two major subtypes: M1 macrophages that work in the inflammatory phase of wound healing; and M2 macrophages that work in the proliferative phase and work to suppress inflammation and restructure the tissues [55, 56]. Both iNOS and IL-6 are inflammatory cytokines that are used as markers for M1 macrophages. The expression of iNOS was significantly higher on PODs 4 and 7 and that of IL-6 was significantly higher on PODs 1 and 4 in the HD-AM group than in the HD-AM (-) group. However, the expression of iNOS and IL-6 tended to decrease on POD 7, after peaking on POD 4, and, in particular, the expression level of IL-6 decreased by about half. On the other hand, the expression of CD163 and IL-10, which are anti-inflammatory cytokines that are used as markers for M2 macrophages, increased over time. CD163 expression increased rapidly on POD 7 and IL-10 expression was significantly enhanced in the HD-AM group on PODs 4 and 7.

When localization was observed by immunohistochemical staining, the expression of Iba1 (an M1 and M2 macrophage marker) and CD163 (an M2 macrophage marker) in the HD-AM group on POD 7 was compared with that of the HD-AM (-) group. From POD 1, M1 macrophages were diffusely observed directly below HD-AM, in fibrin sputum and granulation tissue, whereas many M2 macrophages were observed only in the granulation tissue on POD 7. Recent studies have shown that AM regulates differentiation of M1 to M2 macrophages [30]. HD-AM may have the same function as AM. Early differentiation into M2 macrophages by the HD-AM group is considered an important factor for anti-inflammatory effect and tissue repair.

The expression of TGFβ-1 increased over time in both groups, and the expression was significantly enhanced in the HD-AM group on POD 7. TGF-β1 is a growth factor that has important functions such as proliferation, migration and differentiation of inflammatory cells in the early stages of wound healing [57]. Furthermore, TGFβ-1 is an inhibitory cytokine, expressed on M2 macrophages, that is crucial for homeostasis throughout the body and plays an important role in wound reconstruction. In this study, the high expression of TGF-β1 on POD 7 is considered to reflect the migration of M2 macrophages that exert a powerful anti-inflammatory effect to reduce and terminate the inflammatory response. It has been reported that the AM significantly increases growth factors early in wound healing but acts to suppress it later [58]. Since IL-10 remains in HD-AM, it is considered to exhibit the same action as fresh or cryopreserved AM.

In the presence of HD-AM, on POD 7, the expression of anti-inflammatory cytokines was significantly higher than that of the inflammatory cytokines. As a result of stronger initiation of the inflammatory phase earlier in the wound healing process, it shifted to the proliferative phase more quickly and smoothly. HD-AM autoregulation acted appropriately and effectively in the inflammatory and proliferative phases of the wound healing process. As a result, accumulation of early inflammatory cells (macrophages, neutrophils, T lymphocytes etc.), angiogenesis and the migration and proliferation of fibroblasts were induced. Therefore, we conclude that HD-AM contributed to the earlier formation of good granulation tissue (transplant bed formation).

Recently, it was reported that dehydrated human amniotic and chorionic membrane was effective in treating enterocutaneous fistula, which is a type of infected wound [59], and diabetic foot ulcers [60]. It was suggested that human AM was simple to use and was able to cover large wounds and those with an irregular surface area. No problems were encountered when human AM is used for infected injuries.

Our future aim is to verify the antibacterial effect of HD-AM on *Pseudomonas aeruginosa* and Methicillin-resistant *Staphylococcus Aureus* (MRSA) infections encountered in clinical settings. The clinical implication of this research is to establish a method that takes advantage of the combined effect of vacuum-assisted closure and artificial dermis.

## Conclusion

HD-AM promoted early good granulation growth. The cytokines and scaffolds remaining in HD-AM promoted inflammatory cell infiltration at an early stage good granulation tissue formation with fibroblast proliferation and angiogenesis. Concurrently, it was suggested that neutrophil migration might be promoted and the defense mechanism against the entry of foreign bodies could be enhanced. As a result of HD-AM induction of the inflammatory phase more strongly at an early stage, it was possible to enter the proliferative phase more quickly and smoothly. We believe that the autoregulation of HD-AM during the wound healing process helped to promote good early granulation growth. Therefore, HD-AM is useful as a new wound dressing material for full-thickness skin excision sites after third-degree burn injuries and may be a new therapeutic technique for improving the survival rate of patients with severe burn injuries.

## Data Availability

The datasets used and/or analysed during the current study are available from the corresponding author on reasonable request.

## Abbreviations

TBSA: total body surface area; NPWT: negative-pressure wound therapy; AM: human amniotic membrane; fAM: fresh human amniotic membrane; HD-AM: hyperdry human amniotic membrane; TGF-β1: transforming growth factor beta-1; IL: interleukin; PDGF: platelet-derived growth factor; VEGF: vascular endothelial growth factor; EGF: endothelial growth factor; POD: postoperative day; H&E: hematoxylin–eosin; α-SMA: alpha-smooth muscle actin; mRNA: messenger RNA; qPCR: quantitative polymerase chain reaction; qRT-PCR: quantitative reverse-transcription polymerase chain reaction; iNOS: inducible nitric oxide synthase; COX-2: cyclooxygenase-2; PGE2: prostaglandin E2
